# Exploring the Trade-Off in the Variational Information Bottleneck for Regression with a Single Training Run

**DOI:** 10.3390/e26121043

**Published:** 2024-11-30

**Authors:** Sota Kudo, Naoaki Ono, Shigehiko Kanaya, Ming Huang

**Affiliations:** 1 Graduate School of Science and Technology, Nara Institute of Science and Technology, Ikoma 630-0192, Japan; kudo.sota.ko2@is.naist.jp (S.K.); nono@is.naist.jp (N.O.); skanaya@is.naist.jp (S.K.); 2 Institute of Advanced Computing and Digital Engineering, Shenzhen Institute of Advanced Technology, Shenzhen 518055, China

**Keywords:** information bottleneck, deep learning, regression model, supervised learning

## Abstract

An information bottleneck (IB) enables the acquisition of useful representations from data by retaining necessary information while reducing unnecessary information. In its objective function, the Lagrange multiplier β controls the trade-off between retention and reduction. This study analyzes the Variational Information Bottleneck (VIB), a standard IB method in deep learning, in the settings of regression problems and derives its optimal solution. Based on this analysis, we propose a framework for regression problems that can obtain the optimal solution of the VIB for all β values with a single training run. This is in contrast to conventional methods that require one training run for each β. The optimization performance of this framework is theoretically discussed and experimentally demonstrated. Our approach not only enhances the efficiency of exploring β in regression problems but also deepens the understanding of the IB’s behavior and its effects in this setting.

## 1. Introduction

### 1.1. Information Bottleneck

Information extraction refers to obtaining a new representation by retaining the necessary information from a given input while reducing unnecessary information. An information bottleneck (IB) [1] formalizes this process from an information-theoretic perspective. Consider a source random variable *X* and a target random variable *Y*, with their joint distribution assumed to be known. The objective of the IB is to derive a random variable *Z* from *X* that compresses the information contained in *X* while retaining as much information as possible about *Y*. Formally, with mutual information I(·;·), this can be expressed as follows:

(1) $$\max_{Z\in \Delta }I\left(Z;Y\right)\;s.t.\;I\left(X;Z\right)\leq r$$

Here, Δ represents the set of all random variables *Z* that satisfy the Markov chain Y↔X↔Z. We call this the IB objective. In many practical cases, to avoid constrained optimization, instead of directly optimizing the IB objective, the following objective is used. This is the Lagrangian relaxation [2] of the IB objective and is called the IB Lagrangian [3].

(2) $$L_{IB}\left(Z;\beta \right)=I\left(Z;Y\right)-\beta I\left(X;Z\right)$$

β>0 is called the Lagrange multiplier. The advantage of the IB method lies in its ability to control the trade-off between compression I(X;Z) and prediction I(Z;Y) through β. When β is small, the resulting representation tends to be more predictive, while a larger β leads to a more concise representation.

### 1.2. Methods of IB

Mutual information often involves integrals, which are difficult to calculate, making it challenging to solve the IB Lagrangian in general situations. Consequently, early IB methods were proposed for limited scenarios, such as when *X* and *Y* take on a relatively small number of discrete values [1] or when they follow a joint Gaussian distribution [4]. Later, the Variational Information Bottleneck (VIB) [5] introduced a method to solve the IB Lagrangian in more general situations using variational approximations and deep neural networks (DNNs). This approach has become a standard method of solving IBs in DNN-based applications. The details of the VIB are introduced in Section 3. While this paper focuses on analyzing the VIB, it is important to note that there are other methods for IBs [6,7,8,9,10], as well as other objective functions inspired by IBs [11,12,13,14,15].

### 1.3. Effects and Applications of IB in DNNs

There has been extensive research on the use of IBs in supervised learning. As we will see later, maximizing I(Z;Y) is related to likelihood maximization. In addition to this likelihood maximization, an IB can remove unnecessary information, which can sometimes be detrimental to robustness, through compression. This can have a beneficial effect on deep learning models, which can occasionally become overly flexible. Theoretically, ref. [16] has explored IB-based statistical learning theory using DNNs, and [17] has provided a generalization gap between true mutual information and empirical mutual information in probabilistic models. In particular, the IB framework has shown many experimental benefits in classification problems with DNNs. These include improvements in generalization performance [5,8], invariance to nuisance factors [18], robustness against adversarial attacks [5,8,9], out-of-distribution detection [9,19], domain generalization [20,21], and calibration [19]. There are some works that have applied IBs in regression tasks [6,10,22,23,24]. Ngampruetikorn and Schwab [22] studied the quantification of overfitting in the context of IBs specifically in regression tasks with a linear data generation process. Recently, ref. [10] proposed a superior IB method for regression tasks using Cauchy–Schwarz divergence. While the specific effects of compression in regression problems have not been explored as extensively as in classification, this area holds promise for future research. In addition to supervised learning, the IB has also been used in unsupervised learning [25,26] and as a tool to understand DNNs [27,28,29].

### 1.4. Efficient β Exploration

In general, the optimal value of β to be used during training is not known beforehand. This is because the optimal trade-off between prediction and compression, which maximizes generalization, depends on the task-specific distribution [17]. Therefore, practitioners usually follow a procedure whereby they train multiple models with varying β values and select the model with the best properties. However, the drawback of this approach is that it requires multiple training runs and is computationally expensive.

To make this search more efficient, several studies have been conducted. Ref. [30] showed, that even within the range β∈(0,1), inappropriate values of β can render the IB Lagrangian unlearnable (i.e., the optimal *Z* becomes independent of *X*). They theoretically established sufficient conditions for β to be learnable and proposed an algorithm to estimate the range of β values that are likely to be learnable. This research is valuable in narrowing down the range of β values to be explored from the perspective of the search efficiency. However, finding a useful β still requires multiple training runs. Ref. [31] developed a method to achieve the desired compression rate *r* in a single training run by establishing a one-to-one mapping between β and *r*. However, the challenge of identifying the appropriate compression rate ultimately still requires trial and error. Additionally, ref. [9] used supervised disentangling to obtain a maximally compressed representation *Z* from the training data without reducing I(Z;Y). Although this training process does not depend on β, it is important to note that this representation is not necessarily the most useful. This is because our goal is often to maximize the true I(Z;Y), not the empirical I(Z;Y) observed in the training data. In fact, similar to the bias–variance trade-off, the true I(Z;Y) is determined by the trade-off between the empirical I(Z;Y) and I(X;Z) in the training data [17]. Previously, we proposed a framework called the Flexible Variational Information Bottleneck (FVIB) [32] that enables the VIB objective to be learned for all values of β in a single training run for classification tasks. The FVIB allows models with all β values to be obtained without requiring additional parameters or training time, as compared to a single training process for the VIB, thereby enabling efficient β exploration.

### 1.5. Contributions and the Structure of This Paper

In this paper, we propose FVIB-Regression (FVIB-R) for regression tasks, which allows the VIB objective to be learned for all β values in a single training run. First, in Section 3, we derive the closed-form solution for the VIB in regression tasks. This provides insights into the behavior of the VIB in regression problems and is expected to be useful for future theoretical analysis and improvements to the IB or VIB in this setting. Next, in Section 4, we use this analysis to design FVIB-R and theoretically discuss its optimization performance. Finally, in Section 5.1.1, we experimentally demonstrate the optimization performance of FVIB-R.

## 2. Related Work

### 2.1. Theory of IB

This study analytically derives the optimal solution of the VIB, and its findings have the potential to contribute to the theoretical understanding of both the VIB and IB in the future. Therefore, we discuss the relationship between this work and previous theoretical studies on IBs. Ref. [33] discovered that, in IBs, when β is varied, there is a point at which a significant change in the prediction accuracy occurs, which they referred to as the IB phase transition. They also demonstrated that new correlations are learned during this phase transition. Our study elucidates the behavior of the VIB optimal solution concerning β, which could potentially connect with the theoretical research on the IB phase transition. Kolchinsky et al. [34] studied IBs in the settings of stochastic models and deterministic scenarios. While our research is constrained to regression problems, it does not rely on the deterministic scenario constraint, meaning that it also addresses datasets with multiple different labels for the same input data. Amjad and Geiger [35] theoretically demonstrated several issues when training classification problems using IBs with deterministic DNNs, and they pointed out that stochastic DNNs such as the VIB can avoid these problems. In contrast, our study focuses on the VIB in the context of regression problems. There are many works that study the relationship between information-theoretic quantities and generalization [16,36,37,38,39,40,41,42,43]. In particular, ref. [16] explored statistical learning theory using mutual information in the context of deterministic deep neural networks. Ref. [17] provided a generalization gap between the true mutual information and the empirical mutual information in stochastic models. This research confirms that the VIB is effective for generalization as a stochastic model.

### 2.2. Variational Autoencoders

Variational autoencoders (VAE) [44] and β-VAE [45] have loss functions that include both distortion (prediction) and rate (compression) terms, and they can be interpreted as special cases of the VIB [5,46,47]. Therefore, our analysis is related to the existing analyses of VAE. There have been many studies conducted to understand the characteristics of the optimal solutions in β-VAE [48,49,50]. Our analysis revisits these studies in the context of the VIB, which is a supervised learning framework. By doing so, our analysis extends the insights gained from VAE to the domain of IBs and has the potential to provide a profound understanding of how the interplay between distortion and rate terms can be leveraged in supervised learning scenarios.

## 3. Analysis of VIB in Regression Tasks

In this section, we analyze the VIB in the settings of regression tasks. We begin by explaining the VIB, followed by the introduction of a specific model setup for our analysis. Finally, we derive the optimal solution for the VIB with this model.

### 3.1. Variational Information Bottleneck

The VIB enables the learning of IBs in general settings by providing a lower bound on Equation (2) through variational approximation. When predicting *Y* from *X*, the random variable *Z* is obtained via the feature extractor $p_{\theta }\left(Z|X\right)$. First, let us consider the prediction term, i.e., the first term in Equation (2). By using a new model $q_{\phi }\left(Y|Z\right)$ as a variational approximation of $p_{\theta }\left(Y|Z\right)$, the following variational lower bound is obtained:

(3) $$\begin{array}{cc} I\left(Z;Y\right)\geq \int dx\;dy\;dz\;p\left(x,y\right)\;p_{\theta }\left(z|x\right) & \log q_{\phi }\left(y|z\right)+H\left(Y\right) \end{array}$$

Here, H(Y) represents the entropy of *Y* (or the differential entropy in the case of a regression problem). Estimating H(Y) from observed data is not a straightforward task when *Y* is a continuous variable. However, since this value remains constant throughout the learning process, it can be ignored during training [51]. Next, regarding the compression term I(X;Z) in Equation (2), by using r(Z) as a variational approximation of $p_{\theta }\left(Z\right)$, an upper bound can be obtained:

(4) $$I\left(X;Z\right)\leq \int dx\;p\left(x\right)\;D_{KL}[p_{\theta }\left(Z|x\right)\left|\right|r\left(Z)\right]$$

In practice, a fixed distribution is used for r(Z). By combining these elements, a lower bound on the IB Lagrangian can be obtained. Given data {$\left(x_{1},y_{1}\right),\cdots ,\left(x_{N},y_{N}\right)$}, and by using the empirical distribution as the joint distribution of *X* and *Y*, the objective function of the VIB is derived:

(5) $$\begin{array}{cc} L_{VIB}\left(\theta ,\phi ;\beta \right):= & \frac{1}{N}\sum_{i=1}^{N}\left[E_{p_{\theta }\left(Z|x_{i}\right)}[\log q_{\phi }\left(y_{i}|Z)\right]-\beta D_{KL}[p_{\theta }\left(Z|x_{i}\right)\left|\right|r\left(Z)\right]\right] \end{array}$$

The first term, which is the prediction term, corresponds to the expected value of the log-likelihood, revealing the relationship between maximizing I(Z;Y) and maximum likelihood estimation.

### 3.2. Model Settings for Analysis

Below, the model setup for the analysis is introduced. All subsequent lemmas and theorems are based on the following setup. Consider a *d*-dimensional regression task, using input data x∈X and the continuous label $y\in R^{d}$. The random variable *Z* is defined as $z\in R^{\kappa }$ with κ≥d. We define $p_{\theta }\left(Z|x\right)$ as follows:

(6) $$p_{\theta }\left(Z|x\right)=N(\mu (x),\Sigma (x))$$

Here, let $\mu :X\to R^{\kappa }$ and $\Sigma :X\to \{A\in R^{\kappa \times \kappa }|A≻0\}$, where Σ represents the covariance matrix. Although the covariance matrix is typically restricted to a diagonal matrix, our analysis does not impose this restriction. Based on these settings, the parameters are defined as θ={μ,Σ}. The distribution r(Z) is typically set as r(Z)=N(0,I), and we adopt this in our study as well.

The predictor $q_{\phi }$ is often composed of a single fully connected layer in practice. With this in mind, in our analysis, we define the fully connected layer as v(z):=Wz+b, where $W\in R^{d\times \kappa }$ and $b\in R^{d}$. Using this, $q_{\phi }$ is defined as follows:

(7) $$q_{\phi }\left(y|z\right)=N(v\left(z\right),\frac{1}{2}I)$$

This setting corresponds to the mean squared error (MSE):

(8) $$\log q_{\phi }\left(y|z\right)=-||{v(z)-y||}_{2}^{2}-\frac{d}{2}\log\pi$$

Therefore, ϕ={W,b}. It should be noted that the second term is constant during training.

### 3.3. The Optimal Solution

Under the above settings, we analyze the VIB objective. To this end, we introduce several new definitions. We split the data indices into equivalence classes defined by

(9) $$[i]:=\{j\in \{1,2\cdots ,N\}|x_{j}=x_{i}\}$$

This means that we are grouping data indices with the same input into a single set. Using this, for each label, we reassign the average of the labels whose indices belong to the same equivalence class:

(10) $${\tilde{y}}_{i}:=\frac{1}{\left|[i]\right|}\Sigma_{j\in [i]}y_{j}$$

We define the mean and the covariance matrix for ${\tilde{y}}_{i}$:

(11) $$m_{\tilde{y}}:=\frac{1}{N}\sum_{i=1}^{N}{\tilde{y}}_{i}$$

(12) $$S_{\tilde{y}}:=\frac{1}{N}\sum_{i=1}^{N}({\tilde{y}}_{i}-m_{\tilde{y}})({{\tilde{y}}_{i}-m_{\tilde{y}})}^{⊤}$$

Additionally, in the following, diag(a) denotes a diagonal matrix with the vector a as its diagonal elements, and ${\left[a_{i}\right]}_{i}$ represents a vector with its *i*-th component being $a_{i}$. Using these definitions, we derive the optimal solution for the VIB objective in the context of regression tasks.

**Lemma** **1.** *Consider the model settings in Section 3.2. For any β>0, the feature dimension κ≥d does not affect the value of $\max_{\theta ,\phi }L_{VIB}\left(\theta ,\phi ;\beta \right)$. Below, we consider the case where κ=d. For any β>0, if the parameters satisfy the following conditions, $L_{VIB}\left(\theta ,\phi ;\beta \right)$ is maximized.*

- *$\mu \left(x_{i}\right)=\frac{2}{\beta }({I+\frac{2}{\beta }W^{⊤}W)}^{-1}W^{⊤}({\tilde{y}}_{i}-m_{\tilde{y}})$ for all i=1,2⋯,N.*
- *$\Sigma (x_{i})=({I+\frac{2}{\beta }W^{⊤}W)}^{-1}$ for all i=1,2⋯,N.*
- *$W=\mathrm{Pdiag}([{\pm \sqrt{\max(0,\lambda_{i}-\frac{\beta }{2})}]}_{i})R$, where $S_{\tilde{y}}=:\mathrm{Pdiag}([{\lambda_{i}]}_{i})P^{⊤}$ by orthogonal diagonalization and R is an arbitrary orthogonal matrix.*
- *$b=m_{\tilde{y}}$.*

Refer to Appendix A.1 for the proof. Below, the properties of the optimal solution of the VIB in regression tasks are considered based on Lemma 1. We discuss how well the optimal solution of the VIB optimizes the IB objective, as given by Equation (1). It is important to note that the joint distribution of *X* and *Y* considered here is not the true distribution but the empirical distribution of the training data. The IB framework assumes a Markov chain Y↔X↔Z, and, according to the data processing inequality, we have I(Z;Y)≤I(X;Z). This means that when the IB objective maximizes I(Z;Y) with the limitation of a given I(X;Z), the feasible region is constrained by I(Z;Y)≤I(X;Z). Furthermore, the equality holds if and only if I(X;Z|Y)=0. Now, consider Lemma 1. We see that the representation *Z* in the optimal solution becomes determined solely by *Y* and is conditionally independent of *X* given *Y*, when *X* and *Y* lie within the support of the empirical distribution. This implies I(X;Z|Y)=0, and thus I(Z;Y)=I(X;Z) is achieved. This indicates that the set of optimal solutions for the VIB in the regression tasks includes, at the very least, the optimal solutions of the IB objective under the empirical distribution. This is particularly interesting when considering that the VIB is designed through Lagrangian relaxation and the variational approximation of the original IB objective.

Furthermore, as we show in Section 4, FVIB-R achieves the condition of Lemma 1 through training, thereby reaching the optimal solutions not only of the VIB objective but also of the IB objective.

## 4. Methods

### 4.1. FVIB-R

Based on the above analysis, we design a framework called FVIB-R, which allows the optimization of the VIB objective for all values of β in a single training run for regression problems. In this section, we first introduce the model structure and objective function of FVIB-R. Then, we theoretically discuss FVIB-R’s ability to maximize the VIB objective.

For FVIB-R, we train a model $h_{\psi }:X\to R^{d}$ with parameters ψ∈Ψ by maximizing the following objective function:

(13) $$J_{FVIB-R}\left(\psi \right):=-\frac{1}{N}\sum_{i=1}^{N}||{h_{\psi }\left(x_{i}\right)-({\tilde{y}}_{i}-m_{\tilde{y}})||}_{2}^{2}$$

Note that, unlike the VIB objective, this objective function does not depend on β. Next, using the trained $h_{\psi }$, for any β>0, we set the VIB parameters as follows:

- ${\tilde{\mu }}_{\beta ,\psi }\left(x\right):=\frac{2}{\beta }({I+\frac{2}{\beta }W^{⊤}W)}^{-1}W^{⊤}h_{\psi }\left(x\right)$;
- ${\tilde{\Sigma }}_{\beta }\left(x\right):=({I+\frac{2}{\beta }W^{⊤}W)}^{-1}$;
- ${\tilde{W}}_{\beta }:=P\mathrm{diag}({\left[\sqrt{\max(0,\lambda_{i}-\frac{\beta }{2})}\right]}_{i})$, where $S_{\tilde{y}}=:P\mathrm{diag}({\left[\lambda_{i}\right]}_{i})P^{⊤}$ by orthogonal diagonalization;
- $\tilde{b}:=m_{\tilde{y}}$.

As a result, the value of β does not influence the training process and can be adjusted during the evaluation phase. This is in contrast to the VIB framework, where β affects the training setup and cannot be changed afterward.

In the following, we theoretically argue that, despite the structural advantages mentioned above, this setup and objective function can still learn the VIB objective.

**Theorem** **1.** *Consider the model settings in Section 3.2. If $\lim_{t\to \infty }J_{FVIB-R}\left(\psi_{t}\right)=0$, then, for β>0, the sequence $\{{L_{VIB}({\tilde{\mu }}_{\beta ,\psi_{t}},{\tilde{\Sigma }}_{\beta },{\tilde{W}}_{\beta },\tilde{b};\beta )\}}_{t\in N}$ converges to $\max_{\theta ,\phi }L_{VIB}\left(\theta ,\phi ;\beta \right)$ as t→∞.*

This is derived from the fact that FVIB-R with $J_{FVIB-R}\left(\psi \right)=0$ is the optimal solution to the VIB objective, and from the continuity of the VIB objective. Detailed proof is provided in Appendix A.2. This theorem shows that, for any β, when $J_{FVIB-R}\left(\psi \right)$ is sufficiently maximized, the VIB objective approaches its maximum. This characteristic indicates that FVIB-R can learn the VIB for any β, despite being a learning setup that is independent of β. Note that Theorem 1 is valid when $J_{FVIB-R}\left(\psi \right)$ converges to zero. However, in cases where gradient descent reaches a local optimum, or when the training is stopped early to prevent overfitting, $J_{FVIB-R}\left(\psi \right)$ does not strictly converge to zero. Even in such cases, the following properties are beneficial regardless of the value that $J_{FVIB-R}\left(\psi \right)$ reaches.

**Theorem** **2.** *Consider the model settings in Section 3.2 and assume*

(14) $$\{\left[h_{\psi }\left(x_{1}\right)\;h_{\psi }\left(x_{2}\right)\cdots \;h_{\psi }\left(x_{N}\right)]\;|\psi \in \Psi \right\}=R^{d\times N}.$$

*Then, for any β>0,*

(15) $$\min_{\psi :J_{FVIB-R}\left(\psi \right)=\alpha }L_{VIB}({\tilde{\mu }}_{\beta ,\psi },{\tilde{\Sigma }}_{\beta },{\tilde{W}}_{\beta },\tilde{b};\beta )$$

*is monotonically increasing with respect to α.*

**Figure 1:**
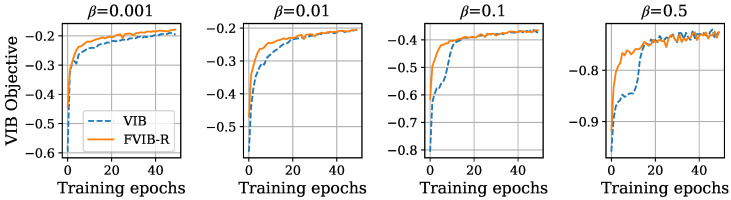
VIB objective values of VIB and FVIB-R during training on California housing prices dataset.

**Figure 2:**
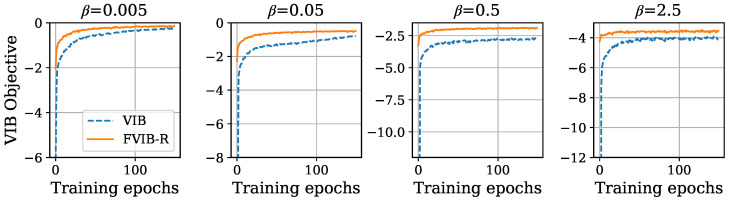
VIB objective values of VIB and FVIB-R during training on Nutrition5k dataset.

Detailed proof can be found in Appendix A.3. The assumption of Equation (14) is justified by the flexibility of neural networks, as shown in the universal approximation theorem [52]. Theorem 2 demonstrates that optimizing $J_{FVIB-R}\left(\psi \right)$ monotonically increases the worst-case value of the VIB objective for any β. In other words, this is a process similar to an increase in a lower bound of the objective function, which is commonly used in practical machine learning applications. It should be noted that this theorem does not strictly guarantee an increase in the VIB objective. However, as seen later in Figure 1 and Figure 2, in the experiments, the VIB objective function does indeed increase. These two theorems together show that the proposed setup and objective function are effective in simultaneously maximizing the VIB objective for all values of β.

### 4.2. Relation to FVIB

Previously, we have proposed the FVIB [32], which enables the learning of the VIB for all values of β in a single training run for classification problems. In this study, we address a similar challenge in the context of regression problems. Here, we will summarize the relationship between FVIB-R and FVIB. Regarding the model setup during the analysis, while the feature extractor $p_{\theta }$ remains the same, the predictor $q_{\phi }$ differs to reflect the problem setting. Specifically, in FVIB-R, $\log q_{\phi }$ is expressed as a quadratic function, allowing the expectation in the prediction term of the VIB objective to be computed analytically. On the other hand, in FVIB, the expectation in the prediction term cannot be solved analytically, and, instead, the Taylor approximation of $\log q_{\phi }$ is used. As a result, FVIB-R directly analyzes the VIB objective, whereas FVIB analyzes an approximation of the VIB objective. Additionally, FVIB assumes a deterministic scenario, where no data points exist with the same *X* but different *Y* values [34]. In contrast, FVIB-R does not require this assumption. This is because, as seen in Equation (A1) in Appendix A.1, any non-deterministic situation in regression problems can be transformed into a deterministic situation without changing the VIB objective by replacing $y_{i}$ with ${\tilde{y}}_{i}$. Overall, for regression tasks, it is possible to analyze the VIB with fewer assumptions and without relying on approximations.

## 5. Experiments

### 5.1. Experimental Setup

We demonstrate the optimization performance of FVIB-R using two real datasets and further investigate its compression effects with a synthetic dataset. Below, we introduce the datasets and the experimental procedure for each setting. The detailed configurations are presented in Table 1.

#### 5.1.1. Real Dataset

The first dataset is the California housing prices dataset [55], which consists of 20,640 samples. This dataset, based on the 1990 California census, involves a regression problem where the goal is to predict a single variable representing house prices using eight source variables, including the latitude, longitude, and the number of rooms. We use the version of the dataset distributed in the scikit-learn package [56]. The second dataset is the Nutrition5k dataset [57]. Nutrition5k contains around 5000 images of food. The task is to predict five continuous variables that indicate calories, mass, and three major nutrients, using these image data as input. The data acquisition and normalization procedures follow [58]. For Nutrition5k, we use parameters pretrained on ImageNet1k [59] as the parameters’ initial values to compensate for the limited data.

The following is an overview of the experimental procedure. We split the data into training, validation, and test sets in a ratio of 80%, 10%, and 10%, respectively. First, for each model, we conduct a grid search over the candidate learning settings, such as the epochs and learning rates, as shown in Table 1. During this step, β is fixed at 0.01 for the California housing prices dataset and 0.05 for the Nutrition5k dataset, and the evaluation metric is the MSE on the validation set. Next, using the best training settings obtained from the grid search, we retrain the models on the training data with $\beta \in \{10^{-6},\cdots ,10^{-2},0.1,0.2,\cdots ,1.0\}$ for the California housing prices dataset and $\beta \in \{5\times 10^{-6},\cdots ,5\times 10^{-2},0.5,1.0,1.5\cdots ,5.0\}$ for the Nutrition5k dataset. Finally, we evaluate these trained models on the test data to assess their performance. All experiments are conducted using PyTorch [60]. All models use a predictor composed of an affine transformation on top of the feature extractor described in Table 1.

#### 5.1.2. Synthetic Dataset

As a synthetic dataset, we sample *x* and *y* from the data generation process represented below:

(16) x∼N(0,1)

(17) y=sin5x+ε

where ε∼N(0,1). For both training and evaluation, 100 samples are used. In experiments that are repeated multiple times, different data samples are used for each trial. To examine the effects of noise, overfitting, and the behavior of compression in response to these factors, a relatively large model is employed for the small dataset. The other settings are shown in Table 1.

### 5.2. Results and Discussion

First, we verify the ability of FVIB-R to optimize the VIB objective. To do this, we compare the VIB objective values across the training epochs between FVIB-R and VIB. Figure 1 and Figure 2 show the VIB objective values for each training epoch on the California housing prices dataset and the Nutrition5k dataset, respectively. It is important to note that the VIB objective values presented here are computed using the training data. While the VIB is trained separately for each value of β, FVIB-R training is conducted only once. In both datasets, the VIB objective values for FVIB-R increase monotonically and converge to values similar or superior to those obtained by directly training the VIB for each β. These results align with the theoretical properties of FVIB-R discussed in Section 4.The reason that FVIB-R outperforms the VIB in optimization for β=0.5 and 2.5 in Figure 2 can be attributed to the fact that the VIB restricts the covariance matrix Σ of *Z* to be diagonal due to computational efficiency constraints, whereas FVIB-R does not impose such a restriction (see Section 3.2). This makes the model more flexible. Additionally, FVIB-R tends to converge faster compared to direct VIB training. This faster convergence might be attributed to the different handling of the expectation of the prediction term in the VIB objective between the VIB and FVIB-R. In the VIB, the expected value is computed based on the sampled *Z*, which introduces fluctuations in the gradient direction. In contrast, FVIB-R analytically computes the expected value and directly learns the optimal solution, avoiding the aforementioned fluctuations in the gradient direction. This difference in the training methods could be the reason for the observed faster convergence in FVIB-R.

**Figure 3:**
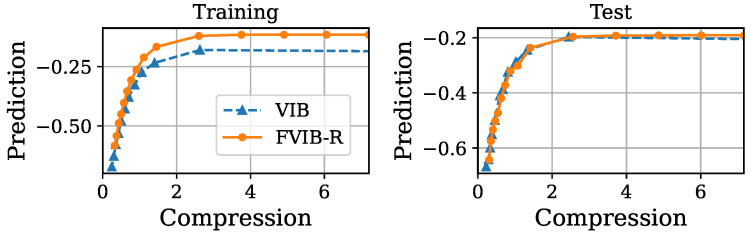
Plots of compression versus prediction obtained by FVIB-R and VIB in California housing prices dataset. Units are nat.

**Figure 4:**
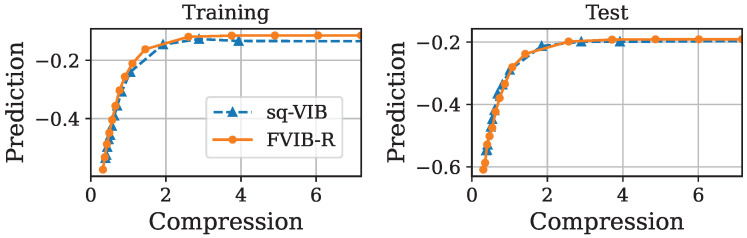
Plots of compression versus prediction obtained by FVIB-R and sq-VIB in California housing prices dataset. Units are nat.

**Figure 5:**
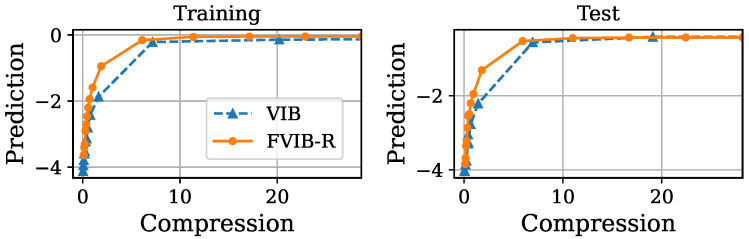
Plots of compression versus prediction obtained by FVIB-R and VIB in Nutrition5k dataset. Units are nat.

**Figure 6:**
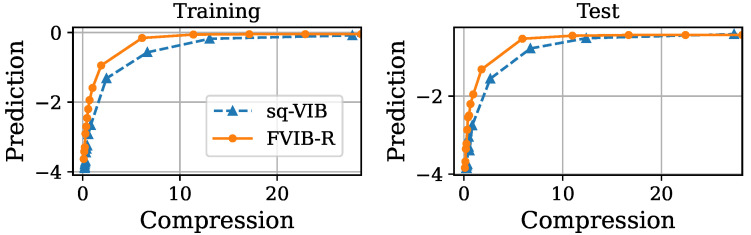
Plots of compression versus prediction obtained by FVIB-R and sq-VIB in Nutrition5k dataset. Units are nat.

Next, we examine FVIB-R’s optimization performance from a different perspective. Figure 3, Figure 4, Figure 5 and Figure 6 illustrate the trade-off between the compression and prediction terms of the VIB objective on the California housing prices dataset and the Nutrition5k dataset, respectively. More precisely, prediction is minus the MSE and compression is the sample mean of the KL divergence in Equation (5). Each point in these plots corresponds to a different value of β. Both the training and test datasets are shown in these figures. Once again, it is important to note that, while the VIB requires multiple training processes for different values of β, FVIB-R is trained only once per dataset. The curve for FVIB-R is shown as a plot due to the evaluation, but the model is able to continuously vary along this curve. In Figure 3 and Figure 4, FVIB-R is compared to the VIB and squared-VIB (sq-VIB) in the California housing prices dataset. The squared versions, proposed by [34], use a squared value for the compression term in the objective function for the purpose of exploring the entire trade-off. In these figures, all methods are evaluated and plotted for $\beta \in \{10^{-6},\cdots ,10^{-2},0.1,0.2,\cdots ,1.0\}$. In Figure 3, we observe that FVIB-R achieves compression–prediction performance on the test data that is comparable to that of the VIB. On the training data, FVIB-R shows better predictions at similar levels of compression compared to the VIB. This difference is likely not due to the framework’s inherent performance, but rather a result of the grid search process selecting models that were either underfitting or overfitting. Moreover, Figure 4 shows that FVIB-R is comparable to sq-VIB both in the training set and in the test set.

Figure 5 and Figure 6 show the optimization performance of FVIB-R compared to the VIB or sq-VIB in the Nutrition5k dataset. In these figures, the VIB and sq-VIB are evaluated for $\beta \in \{5\times 10^{-6},\cdots ,5\times 10^{-2},0.5,1.0,1.5\cdots ,5.0\}$. In Figure 5, FVIB-R demonstrates performance comparable to the VIB’s in both the training and test data. Figure 6 shows that the highest value of prediction for FVIB-R is similar to that of sq-VIB, while FVIB-R is more predictive than sq-VIB when the representation is compressed to some extent. In conclusion, despite the more efficient training process of FVIB-R, its performance is comparable (or sometimes superior) to that of the traditional VIBs on both the training and test data.

Finally, we present the quantitative evaluation of the performance. Here, we show the MSE as it reflects I(Z;Y), as shown in Equations (3) and (8). In addition to the VIB, we compare FVIB-R with the Nonlinear Information Bottleneck (NIB) [6], as well as the squared-VIB (sq-VIB) and squared-NIB (sq-NIB). Table 2 shows the MSE on the test data and the number of parameters required for each IB method. For each method, the best model is selected based on the MSE in the validation set across $\beta \in \{10^{-6},\cdots ,10^{-2},0.1,0.2,\cdots ,1.0\}$ for the California housing prices dataset and $\beta \in \{5\times 10^{-6},\cdots ,5\times 10^{-2},0.5,1.0,1.5\cdots ,5.0\}$ for the Nutrition5k dataset and then evaluated on the test set. In the parameter count notation, the last multiplication indicates the number of models required for the β search. When comparing FVIB-R to the VIB, FVIB-R outperforms the VIB on the California housing prices dataset, while the VIB outperforms FVIB-R on the Nutrition5k dataset. Similarly, when compared to other IB methods, FVIB-R outperforms them on one dataset, while the other methods perform better on the other dataset. Overall, despite reducing the number of parameters, FVIB-R demonstrates competitive performance with the other IB methods. This performance highlights that FVIB-R can achieve comparable results with fewer parameters, making it an efficient alternative to traditional IB methods.

**Figure 7:**
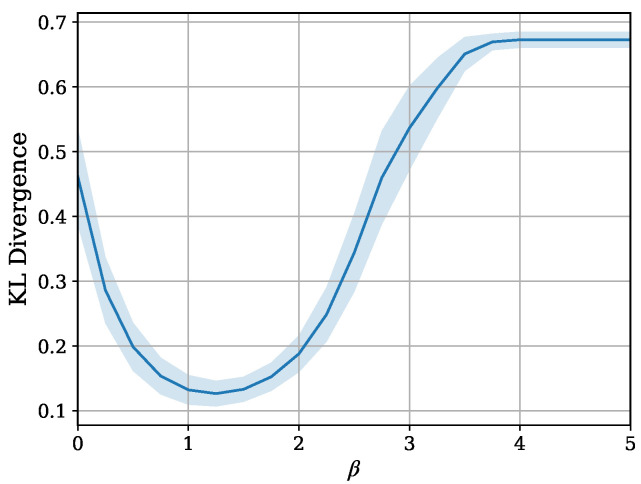
The expected KL divergence between the true *y* and its prediction is computed for each β. A 95% confidence interval, obtained from 30 trials, is also shown. Units are nat.

With the synthetic data, we investigate the distributional difference between the true p(y|x) and its prediction changes for each β. For this purpose, the expected KL divergence, represented below, is utilized.

(18) $$E_{x}[D_{KL}(p\left(y|x\right)||p\left(\hat{y}|x\right))]$$

where $\hat{y}$ is the predicted random variable obtained from $q_{\phi }\left(y|z\right)$. For the estimation, the sample mean of the analytically derived KL divergence is calculated. The results are shown in Figure 7. It is observed that the predicted distribution moves closer to the true distribution with suitable compression.

**Figure 8:**
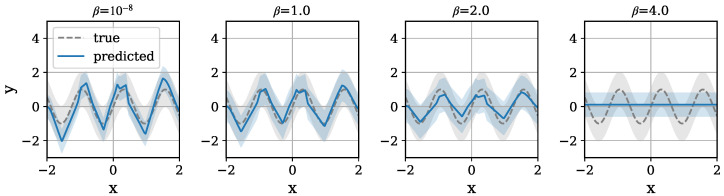
The true distribution of *y* and its prediction given each *x*. The standard deviation is also shown for both distributions. Units are nat.

The distributions of the true p(y|x) and the predicted $p(\hat{y}|x)$ for each β are shown in Figure 8. These results demonstrate that compression leads to a smoother mean line and wider variance. When β=1.0, the predicted distribution is the closest match to the true distribution, which is consistent with the observations in Figure 7. The detailed behavior of compression and its limitations are discussed in the Limitations section.

## 6. Limitations and Future Work

Lemma 1 highlights the potential shortcomings of the VIB in regression problems, while also showing that FVIB-R inherits these shortcomings. The key points are summarized below. The first limitation lies in the simplicity of the compression procedure. Lemma 1 demonstrates the optimal solution of the VIB for regression problems. In particular, μ(x) can be viewed as a combination of $\left({\tilde{y}}_{i}-m_{\tilde{y}}\right)$ and a linear transformation controlled by β. Here, the former is deterministically determined by the data, and thus includes noise, while the latter performs compression through its transformation. However, since this is a simple linear transformation, the noise that can be eliminated is limited. FVIB-R, which is designed based on this, shares the same limitation. Future research could explore comparisons with other IB methods in this regard and investigate more flexible compression approaches. Another limitation lies in the certain divergence from the properties of the IB. It is theoretically known that the IB undergoes phase transitions, and, to the best of our knowledge, the phase transitions of the VIB have been experimentally confirmed in classification problems [33]. However, since Lemma 1 represents the solution of the VIB in regression problems, it suggests that phase transitions may not occur in such cases. This is because the VIB (and FVIB-R) aims to increase the IB’s lower bound for the empirical distribution, and there is no guarantee that they will reach the IB optimal solution for the true distribution. Improvements to the VIB and FVIB-R in this regard will be left for future work.

## 7. Conclusions

In this study, we explore the VIB in the context of regression problems and analytically derive its optimal solution. We anticipate that this analysis will contribute to the theoretical understanding and improvement of both the VIB and the broader IB framework in the future. Using this analysis, we propose a framework that allows the VIB objective to be learned for all values of β in a single training run for regression problems. Additionally, this framework explicitly demonstrates the compression method, enabling a more intuitive understanding. Finally, we theoretically justify the optimization performance of this framework and validate its properties through experiments. This method not only offers direct practical applications but also serves as a valuable tool for an understanding of the behavior and impact of IBs in regression problems. We believe that this framework will facilitate further research and practical advancements in the field of IBs.

## Figures and Tables

**Table 1:** Experimental setup. In the description of the feature extractor, the subscripts of each module indicate the output dimension.

	California Housing Prices	Nutrition5k	Synthetic Dataset
Feature extractor	$x\in R^{8}$	$x\in R^{3\times 480\times 640}$	x∈R
	$\to Linear_{128}$	$\to ResNet18_{\kappa \;or\;2\kappa }$ [53]	$\to Linear_{128}$
	$\to ReLU\to Linear_{128}$		$\to ReLU\to Linear_{128}$
	$\to ReLU\to Linear_{\kappa \;or\;2\kappa }$		$\to ReLU\to Linear_{128}$
			$\to ReLU\to Linear_{128}$
			$\to ReLU\to Linear_{1}$
κ in non-FVIB-R models	32	256	−
# Epochs	{50, 100, 150, 200}	{50, 100, 150, 200}	200
Learning rate	{$1.0\times 10^{-4}$, $1.0\times 10^{-3}$}	{$1.0\times 10^{-4}$, $1.0\times 10^{-3}$}	$1.0\times 10^{-3}$
Optimizer	Adam [54]	Adam	Adam

**Table 2:** Mean squared error and the number of parameters of FVIB-R compared to other IB methods.

	California Housing Prices		Nutrition5k	
Method	MSE	# Parameters	MSE	# Parameters
FVIB-R	0.1910	$1.8\times 10^{4}\times 1$	0.0881	$1.1\times 10^{7}\times 1$
VIB	0.1979	$2.6\times 10^{4}\times 15$	0.0805	$1.1\times 10^{7}\times 15$
sq-VIB	0.1786	$2.6\times 10^{4}\times 15$	0.0893	$1.1\times 10^{7}\times 15$
NIB	0.1816	$2.2\times 10^{4}\times 15$	0.1549	$1.1\times 10^{7}\times 15$
sq-NIB	0.1790	$2.2\times 10^{4}\times 15$	0.1431	$1.1\times 10^{7}\times 15$

## Data Availability

All datasets used in this study are publicly available and described in Section 5.1.1.

## Appendix A

### Appendix A.1. Proof of Lemma 1

**Proof.** We start with the prediction term of the VIB objective. The quotient set is denoted as Λ:={[i]|i∈{1,2⋯,N}}. The step from the second to the third equality uses Equation (8).

(A1) $$\begin{array}{cc} \; & \frac{1}{N}\sum_{i=1}^{N}E_{z∼p_{\phi }\left(Z|x_{i}\right)}[\log q_{\phi }\left(y_{i}|z\right)]\\ = & \frac{1}{N}\sum_{[i]\in \Lambda }\sum_{j\in [i]}E_{z∼p_{\phi }\left(Z|x_{i}\right)}[\log q_{\phi }\left(y_{j}|z\right)]\\ = & \frac{1}{N}\sum_{[i]\in \Lambda }E_{z∼p_{\phi }\left(Z|x_{i}\right)}[\sum_{j\in [i]}-||{v\left(Z\right)-y_{j}||}^{2}]-\frac{d}{2}\log\pi \\ = & \frac{1}{N}\sum_{[i]\in \Lambda }E_{z∼p_{\phi }\left(Z|x_{i}\right)}[\sum_{j\in [i]}-||{v\left(Z\right)-{\tilde{y}}_{j}||}^{2}]+\sum_{j\in [i]}\left({\left||{\tilde{y}}_{j}|\right|}^{2}-{\left||y_{j}|\right|}^{2}\right)-\frac{d}{2}\log\pi \\ = & \frac{1}{N}\sum_{i=1}^{N}E_{z∼p_{\phi }\left(Z|x_{i}\right)}[\log q_{\phi }\left({\tilde{y}}_{i}|z\right)]+{\left||{\tilde{y}}_{i}|\right|}^{2}-{\left||y_{i}|\right|}^{2} \end{array}$$

Furthermore,

(A2) $$\begin{array}{cc} \; & E_{z∼p_{\phi }\left(Z|x_{i}\right)}[\log q_{\phi }\left({\tilde{y}}_{i}|z\right)]\\ = & E_{v∼N(W\mu \left(x_{i}\right)+b,\;W\Sigma \left(x_{i}\right)W^{⊤})}[-{\left||v-{\tilde{y}}_{i}|\right|}^{2}]-\frac{d}{2}\log\pi \\ = & 2{\tilde{y}}_{i}^{⊤}(W\mu (x_{i})+b)-\mathrm{tr}(W\Sigma \left(x_{i}\right)W^{⊤})-{\left||W\mu (x_{i})+b|\right|}^{2}-{\left||{\tilde{y}}_{i}|\right|}^{2}-\frac{d}{2}\log\pi \end{array}$$

The KL divergence in the VIB objective under the model settings in Section 3.2 is represented as

(A3) $$D_{KL}(p_{\theta }(Z|x_{i})\left|\right|r\left(Z\right))=\frac{1}{2}(\mathrm{tr}(\Sigma (x_{i}))-\log|\Sigma (x_{i})|+{\left||\mu (x_{i})|\right|}_{2}^{2}-\kappa )$$

From the above, we obtain

(A4) $$\begin{array}{cc} \; & L_{VIB}\left(\theta ,\phi ;\beta \right)\\ = & \frac{1}{N}\sum_{i=1}^{N}\left[-\frac{\beta }{2}(\mathrm{tr}(\left(I+\frac{2}{\beta }W^{⊤}W)\Sigma (x_{i}\right))+\log|\Sigma {\left(x_{i}\right)}^{-1}|)-\frac{\beta }{2}\mu {\left(x_{i}\right)}^{⊤}(I+\frac{2}{\beta }W^{⊤}W)\mu \left(x_{i}\right)\right.\\ \; & \left.+2({{\tilde{y}}_{i}-b)}^{⊤}W\mu \left(x_{i}\right)-{\left||{\tilde{y}}_{i}-b|\right|}^{2}+{\left||{\tilde{y}}_{i}|\right|}^{2}-{\left||y_{i}|\right|}^{2}-\frac{d}{2}\log\pi +\frac{\beta }{2}\kappa \right] \end{array}$$

Here, we use the following proposition.
*If matrix B is positive definite, then*

(A5)
$$B=\underset{D≻0}{\mathrm{argmin}}(\mathrm{tr}(BD^{-1})+\log|D|)$$

One can refer to Lemma 2 in [50] for the proof. From $I+\frac{2}{\beta }W^{⊤}W≻0$, by maximizing Equation (A4) with respect to $\Sigma (x_{i})$, we obtain

(A6) $$\hat{\Sigma }(x_{i})=({I+\frac{2}{\beta }W^{⊤}W)}^{-1}$$

By completing the square with respect to the quadratic form of $\mu (x_{i})$, we obtain $\mu (x_{i})$ that maximizes the VIB objective.

(A7) $$\hat{\mu }\left(x_{i}\right)=\frac{2}{\beta }\hat{\Sigma }\left(x_{i}\right)W^{⊤}({\tilde{y}}_{i}-b)$$

Here, the above is feasible because the same $\tilde{y}$ is associated with the same input *x* (i.e., a deterministic scenario), from the definition of $\tilde{y}$. From these, we obtain

(A8) $$\begin{array}{cc} \; & L_{VIB}\left(\hat{\mu },\hat{\Sigma },\phi ;\beta \right)\\ = & \frac{1}{N}\sum_{i=1}^{N}\left[({{\tilde{y}}_{i}-b)}^{⊤}(\frac{2}{\beta }W\hat{\Sigma }\left(x_{i}\right)W^{⊤}-I)({\tilde{y}}_{i}-b)-\frac{\beta }{2}\log|\Sigma {\left(x_{i}\right)}^{-1}|+{\left||{\tilde{y}}_{i}|\right|}^{2}-{\left||y_{i}|\right|}^{2}-\frac{d}{2}\log\pi \right] \end{array}$$

Considering singular value decomposition, we have $W=:U\tilde{D}V^{⊤}$, where $U\in R^{d\times d},V\in R^{\kappa \times \kappa }$ are orthogonal matrices. In this case, $\tilde{D}$ is represented as

(A9) $$\tilde{D}=\left[\begin{array}{cccc} \delta_{1} & \; & 0 & \\ & \ddots & & 0\\ 0 & \; & \delta_{d} & \end{array}\right]\in R^{d\times \kappa }$$

We have

(A10) $$\begin{array}{c} \hat{\Sigma }\left(x_{i}\right)=({V(I+\frac{2}{\beta }{\tilde{D}}^{⊤}\tilde{D})V^{⊤})}^{-1}=V\hat{D}V^{⊤} \end{array}$$

where

(A11) $$\hat{D}:=\mathrm{diag}(\frac{1}{1+2\beta^{-1}\delta_{1}^{2}},\cdots ,\frac{1}{1+2\beta^{-1}\delta_{d}^{2}},1,\cdots ,1)\in R^{\kappa \times \kappa }$$

Then, we calculate $\frac{2}{\beta }W\hat{\Sigma }\left(x_{i}\right)W^{⊤}-I$ and $\log|\hat{\Sigma }{\left(x_{i}\right)}^{-1}|$ in Equation (A8) as follows.

(A12) $$\begin{array}{cc} \frac{2}{\beta }W\hat{\Sigma }\left(x_{i}\right)W^{⊤}-I & =\frac{2}{\beta }U\tilde{D}\hat{D}{\tilde{D}}^{⊤}U^{⊤}-I\\ \; & =-U(-\frac{2}{\beta }\tilde{D}\hat{D}{\tilde{D}}^{⊤}+I)U^{⊤}\\ \; & =-U({-\frac{2}{\beta }\tilde{D}{\tilde{D}}^{⊤}+I)}^{-1}U^{⊤}\\ \; & =-\frac{\beta }{2}({WW^{⊤}+\frac{\beta }{2}I)}^{-1} \end{array}$$

(A13) $$\begin{array}{cc} \log|\hat{\Sigma }{\left(x_{i}\right)}^{-1}| & =\log|I+\frac{2}{\beta }{\tilde{D}}^{⊤}\tilde{D}|\\ \; & =\log|I+\frac{2}{\beta }\tilde{D}{\tilde{D}}^{⊤}|\\ \; & =\log|U(I+\frac{2}{\beta }\tilde{D}{\tilde{D}}^{⊤})U^{⊤}|\\ \; & =\log|WW^{⊤}+\frac{\beta }{2}I|+d\log\frac{2}{\beta } \end{array}$$

Substituting Equations (A12) and (A13) into Equation (A8), we have

(A14) $$L_{VIB}\left(\hat{\mu },\hat{\Sigma },\phi ;\beta \right)=-\frac{\beta }{2}(\frac{1}{N}\sum_{i=1}^{N}({{\tilde{y}}_{i}-b)}^{⊤}C^{-1}({\tilde{y}}_{i}-b)+\log|C|)+a$$

where

(A15) $$C:=WW^{⊤}+\frac{\beta }{2}I\in R^{d\times d}$$

and *a* is a constant against the parameters and κ. Since $C^{-1}$ is positive definite,

(A16) $$\hat{b}:=\frac{1}{N}\sum_{i=1}^{N}{\tilde{y}}_{i}=m_{\tilde{y}}$$

and we maximize the objective with respect to b:

(A17) $$L_{VIB}\left(\hat{\mu },\hat{\Sigma },\hat{b},W;\beta \right)=-\frac{\beta }{2}(\mathrm{tr}(C^{-1}S_{\tilde{y}})+\log|C|)+a$$

Finally, we consider the optimal *W*. While the representation above indicates that this is the same situation as in the maximum likelihood estimate of probabilistic PCA [61], we introduce another method to find it. We utilize Ruhe’s trace inequality.
*Let A and B be n×n positive semidefinite matrices and $\lambda_{i}$ be a function that maps a matrix to its i-th smallest eigenvalue. Then,*

(A18)
$$\mathrm{tr}\left(AB\right)\geq \sum_{i=1}^{n}\lambda_{i}\left(A\right)\lambda_{n-i+1}\left(B\right)$$

Refer to H.1.h. in [62] for the proof. Using orthogonal diagonalization, we denote $S_{\tilde{y}}=:P\mathrm{diag}(\lambda_{1},\lambda_{2}\cdots ,\lambda_{d})P^{⊤}$, with $0\leq \lambda_{1}\leq \lambda_{2}\cdots \leq \lambda_{d}$. Without loss of generality of *W*, we assume $|\delta_{1}\left|\leq \right|\delta_{2}\left|\cdots \leq \right|\delta_{d}|$ in Equation (A9). We find the optimal *U*, $\delta_{i}$ and *V* to derive the optimal *W*.

(A19) $$C=U\mathrm{diag}(\left[{\frac{\beta }{2}+\delta_{i}^{2}]}_{i}\right)U^{⊤}$$

*C* is independent of *V*, indicating that *V* does not affect the objective.

(A20) $$\begin{array}{cc} \log|C| & =\log\Pi_{i=1}^{d}(\frac{\beta }{2}+\delta_{i}^{2})\\ \; & =\sum_{i=1}^{d}\log(\frac{\beta }{2}+\delta_{i}^{2}) \end{array}$$

Since log|C| is independent of *U*, it is sufficient to consider minimizing $\mathrm{tr}(C^{-1}S_{\tilde{y}})$ for the optimal *U*. Using Ruhe’s trace inequality, below, it is shown that, when U=P, the trace is minimized. When U=P,

(A21) $$\begin{array}{cc} \mathrm{tr}(C^{-1}S_{\tilde{y}}) & =\mathrm{tr}(U\mathrm{diag}\left([{\left({\frac{\beta }{2}+\delta_{i}^{2})}^{-1}\right]}_{i}\right)U^{⊤}P\mathrm{diag}(\left[{\lambda_{i}]}_{i}\right)P^{⊤})\\ \; & =\sum_{i=1}^{d}\frac{\lambda_{i}}{\frac{\beta }{2}+\delta_{i}^{2}} \end{array}$$

This is the equality of Ruhe’s trace inequality. In this case, the objective is

(A22) $$L_{VIB}\left(\hat{\mu },\hat{\Sigma },\hat{b},U=P,{\left[\delta_{i}\right]}_{i};\beta \right)=-\frac{\beta }{2}\sum_{i=1}^{d}(\frac{\lambda_{i}}{\frac{\beta }{2}+\delta_{i}^{2}}+\log(\frac{\beta }{2}+\delta_{i}^{2}))+a$$

Here, consider a function $f\left(w\right):=\frac{\lambda }{w}+\log w$ for w>0. This function monotonically decreases for w≤λ, achieving the minimum at w=λ, and monotonically increases for w≥λ. Given this, we can find the optimal δ as follows. If $\lambda_{i}>\frac{\beta }{2}$, maximizing the objective with respect to $\delta_{i}$ requires $\frac{\beta }{2}+\delta_{i}^{2}=\lambda_{i}$, indicating that $\delta_{i}=\pm \sqrt{\lambda_{i}-\frac{\beta }{2}}$. If $\lambda_{i}\leq \frac{\beta }{2}$, $\delta_{i}=0$ maximizes the objective. In summary,

(A23) $$\delta_{i}=\pm \sqrt{\max(0,\lambda_{i}-\frac{\beta }{2})}$$

maximizes the objective, and this meets the assumption of $|\delta_{1}\left|\leq \right|\delta_{2}\left|\cdots \leq \right|\delta_{d}|$. Even if the representation of $S_{\tilde{y}}=:P\mathrm{diag}(\lambda_{1},\lambda_{2}\cdots ,\lambda_{d})P^{⊤}$ does not satisfy $0\leq \lambda_{1}\leq \lambda_{2}\cdots \leq \lambda_{d}$, setting U=P and Equation (A23) result in the same objective value. Additionally, substituting Equation (A23) into Equation (A22) shows that $\max_{\theta ,\phi }L\left(\theta ,\phi ;\beta \right)$ is independent of the value of κ≥d. Thus, we consider the case where κ=d. From the above, the optimal *W* is

(A24) $$W=P\mathrm{diag}([{\pm \sqrt{\max(0,\lambda_{i}-\frac{\beta }{2})}]}_{i})R$$

where $S_{\tilde{y}}=:P\mathrm{diag}([{\lambda_{i}]}_{i})P^{⊤}$ by orthogonal diagonalization and *R* is an arbitrary orthogonal matrix. □

### Appendix A.2. Proof of Theorem 1

**Proof.** From Equation (A4), we obtain

(A25) $$\begin{array}{cc} \; & L_{VIB}({\tilde{\mu }}_{\psi ,\beta },{\tilde{\Sigma }}_{\beta },{\tilde{W}}_{\beta },\tilde{b};\beta )\\ = & -\frac{2}{N\beta }\sum_{i=1}^{N}[({h_{\psi }\left(x_{i}\right)-({\tilde{y}}_{i}-m_{\tilde{y}}))}^{⊤}{\tilde{W}}_{\beta }{\tilde{\Sigma }}_{\beta }\left(x_{i}\right){\tilde{W}}_{\beta }^{⊤}(h_{\psi }\left(x_{i}\right)-({\tilde{y}}_{i}-m_{\tilde{y}}))]\\ \; & +g_{\beta }({\tilde{\Sigma }}_{\beta },{\tilde{W}}_{\beta },\tilde{b}) \end{array}$$

where $g_{\beta }$ is a function of Σ, *W* and b, meaning that it is independent of $h_{\psi }\left(x_{i}\right)$. From Lemma 1, substituting ${\tilde{y}}_{i}-m_{\tilde{y}}$ into $h_{\psi }\left(x_{i}\right)$ in Equation (A25) results in $\max_{\phi ,\theta }L_{VIB}\left(\phi ,\theta ;\beta \right)$. Thus,

(A26) $$\begin{array}{cc} \; & L_{VIB}({\tilde{\mu }}_{\beta ,\psi_{t}},{\tilde{\Sigma }}_{\beta },{\tilde{W}}_{\beta };\beta )-\max_{\phi ,\theta }L_{VIB}\left(\phi ,\theta ;\beta \right)\\ = & -\frac{2}{N\beta }\sum_{i=1}^{N}[({h_{\psi_{t}}\left(x_{i}\right)-({\tilde{y}}_{i}-m_{\tilde{y}}))}^{⊤}{\tilde{W}}_{\beta }{\tilde{\Sigma }}_{\beta }\left(x_{i}\right){\tilde{W}}_{\beta }^{⊤}(h_{\psi_{t}}\left(x_{i}\right)-({\tilde{y}}_{i}-m_{\tilde{y}}))] \end{array}$$

If $\lim_{t\to \infty }J_{FVIB-R}\left(\psi_{t}\right)=0$, then, for all i=1,2⋯,N, $h_{\psi_{t}}\left(x_{i}\right)\to ({\tilde{y}}_{i}-m_{\tilde{y}})$, indicating that $L_{VIB}({\tilde{\mu }}_{\beta ,\psi_{t}},{\tilde{\Sigma }}_{\beta },{\tilde{W}}_{\beta };\beta )\to \max_{\phi ,\theta }L_{VIB}\left(\phi ,\theta ;\beta \right)$ as t→∞. □

### Appendix A.3. Proof of Theorem 2

**Proof.** Let $h_{\psi ,all}\in R^{dN}$ be the vector concatenating $h_{\psi }\left(x_{i}\right)$, i.e., $h_{\psi ,all}:=[h_{\psi }{\left(x_{1}\right)}^{⊤}\cdots$$h_{\psi }{\left(x_{N}\right)}^{⊤}{]}^{⊤}$, and let ${\hat{h}}_{all}$ be the vector formed similarly by concatenating ${\hat{h}}_{i}:=({\tilde{y}}_{i}-m_{\tilde{y}})$. For β>0, ${\tilde{W}}_{\beta }{\tilde{\Sigma }}_{\beta }\left(x_{i}\right){\tilde{W}}_{\beta }^{⊤}$ is positive semidefinite. Thus, from Equation (A25), for β>0, $L_{VIB}({\tilde{\mu }}_{\beta ,\psi },{\tilde{\Sigma }}_{\beta },{\tilde{W}}_{\beta };\beta )$ is concave as a function of $h_{\psi ,all}$ and its global optimal solution is ${\hat{h}}_{all}$. Furthermore, $J_{FVIB-R}\left(\psi \right)=-\frac{1}{N}{\left||h_{\psi ,all}-{\hat{h}}_{all}|\right|}_{2}^{2}$, and, given the assumption, $\{h_{\psi ,all}|\psi \in \Psi \}=R^{dN}$. From the above, we need to show the following.
*Let $f:R^{n}\to R$ be convex with $\hat{x}\in R^{n}$ as its global optimal solution. If $0\leq a_{1}<a_{2}$, then $max_{x\in R^{n}:||x-\hat{x}||=a_{1}}f\left(x\right)\leq max_{x\in R^{n}:||x-\hat{x}||=a_{2}}f\left(x\right)$.*
For $a_{1}=0$, this statement holds true immediately. Now, consider the case where $a_{1}>0$. For any $x_{1}$ such that $||x_{1}-\hat{x}||=a_{1}$, define

(A27) $$x_{2}=\hat{x}+\frac{a_{2}}{a_{1}}\left(x_{1}-\hat{x}\right)$$

and $||x_{2}-\hat{x}||=a_{2}$. Let $\lambda :=\frac{a_{1}}{a_{2}}$; then, 0<λ<1 and

(A28) $$x_{1}=\lambda x_{2}+\left(1-\lambda \right)\hat{x}$$

Since *f* is convex,

(A29) $$f\left(x_{1}\right)\leq max\{f\left(x_{2}\right),f\left(\hat{x}\right)\}=f\left(x_{2}\right)$$

Hence, the assertion is proven. □
